# Prevalence of prenatal depression and associated factors among HIV-positive women in primary care in Mpumalanga province, South Africa

**DOI:** 10.1080/17290376.2016.1189847

**Published:** 2016-06-02

**Authors:** Karl Peltzer, Violeta J. Rodriguez, Deborah Jones

**Affiliations:** ^a^ PhD, is a distinguished Research Fellow in HIV/AIDS/STIs and TB (HAST) Research Programme, Human Sciences Research Council, Pretoria, South Africa; ^b^ is a Professor at the ASEAN Institute for Health Development, Mahidol University, Salaya, Thailand; ^c^ is a Research Associate in the Department of Research & Innovation, University of Limpopo, Sovenga, South Africa; ^d^ MSEd, is a Research Associate in Department of Psychiatry & Behavioral Sciences, University of Miami Miller School of Medicine, Miami, USA; ^e^ PhD, is a Professor in Department of Psychiatry & Behavioral Sciences, University of Miami Miller School of Medicine, Miami, USA

**Keywords:** *antenatal care*, *HIV positive*, *pregnant women*, *depressive symptoms*, *Edinburgh Postnatal Depression Scale 10*, *South Africa*, Des soins prénataux, VIH positive, femmes enceintes, symptôme dépressifs, Edinburgh Postnatal Depression Scale 10, Afrique du Sud

## Abstract

This study aimed to assess the prevalence of depressed symptoms and associated factors in prenatal HIV-positive women in primary care facilities in rural South Africa. In a cross-sectional study, 663 HIV-positive prenatal women in 12 community health centres in Mpumalanga province, South Africa, were recruited by systematic sampling (every consecutive patient after HIV post-test counselling). Results indicate that overall, 48.7% [95% CI: 44.8, 52.6] of women during the prenatal period reported depressed mood (scores of ≥ 13 on the Edinburgh Postnatal Depression Scale 10). In multivariate analysis, not being employed, unplanned pregnancy, not having an HIV-positive child, poor antiretroviral therapy adherence, non-condom use at last sex, and intimate partner violence were associated with depressive symptoms. Potential risk factors among HIV-infected prenatal women were identified which could be utilized in interventions. Routine screening for depression may be integrated into prenatal care settings.

## Résumé

Cette étude a pour but de démontrer la prévalence de symptômes dépressifs et d'autres facteurs associés chez des femmes prénatales infectées par le VIH. Les femmes ont été évaluées lors des consultations médicales d'Attention Primaire dans des entourages ruraux d'Afrique du Sud. L'étude transversale a été menée dans 12 Centres de Santé de la province de Mpumalanga avec 663 femmes prénatales, positives au VIH. Les femmes ont été recrutées par échantillonnage systématique (chaque patiente après conseil suivant le résultat du test VIH). Les résultats d'ensemble montrent que le 48.7% des femmes dans la période prénatale [95% IC: 44.8, 52.6] ont manifesté un état animique dépressif (chiffre >=13 d'après the Edinburgh Postnatal Depression Scale 10). Une analyse multivariée (multivariate analysis), pas réalisée, aurait confirmé comme des facteurs associés avec les symptômes dépressifs, la prégnance non planifiée, la peur d'avoir un enfant positive au VIH, une mauvaise adhésion au traitement ART, pas d'utilisation du condom lors de la dernière relation sexuelle, et violence intime du partenaire. Des facteurs potentiels de risque chez des femmes prénatales infectées par le VIH ont été identifiés, ils pourraient être utilisés dans des interventions. Dans ce sens, des études de routine pour dépression pourraient être incorporées dans des protocoles des soins prénataux.

## Introduction

Prenatal depression and anxiety have been associated with increased risk of preterm birth and low birth weight, and delayed cognitive and language development, behavioural and emotional problems (Smith, Shao, Howell, Lin & Yonkers 2011; Van den Berg, Mulder, Mennes & Glover 2005). Infants of depressed mothers are less playful, exhibit less positive affect and are more irritable than infants of non-depressed mothers (Goodman & Brand 2009; O’Hara 2009).

Several studies have investigated the prevalence of depressive symptoms among HIV-positive pregnant women, 42% in Tanzania (Smith Fawzi, Kaaya, Mbwambo, Msamanga, Antelman, Wei, *et al*. 2007), 39% and 52.9% in Uganda (Kaida, Matthews, Ashaba, Tsai, Kanters, Robak, *et al*. 2014), 78% in Thailand (Ross, Sawatphanit & Zeller 2009), and 85% in Zambia (Kwalombota 2002). Some studies show a higher rate of depressive symptoms in HIV-positive pregnant women than in HIV negative pregnant women (Manikkam & Burns 2012; Natamba, Achan, Arbach, Oyok, Ghosh, Mehta, *et al*. 2014), while other studies do not find such a difference (Bonacquisti, Geller & Aaron 2014; Rochat, Richter, Doll, Buthelezi, Tomkins & Stein, 2006). In a recent review, it was concluded that depression, as well as other psychiatric symptomatology, among women living with HIV can adversely affect psychological health and quality of life, as well as clinical outcomes (Kapetanovic, Dass-Brailsford, Nora & Talisman 2014). Kapetanovic *et al*. (2014) further argue that the increasing number of HIV-infected women reaching reproductive age and greater accessibility to protocols aimed at Preventing Mother-to-Child Transmission of HIV (MTCT), highlight a greater need to explore how mental health-related outcomes may affect women’s quality of life and reproductive health, and increase the risk for vertical transmission of HIV.

Many factors associated with depressive symptoms in HIV-positive pregnant women and pregnant women in high HIV epidemic regions have been identified. Some of the sociodemographic factors reported include younger age (Hartley, Tomlinson, Greco, Comulada, Stewart, le Roux, *et al*. 2011), having any marital status other than ‘never married’ (Kaida *et al*. 2014), single marital status (Manikkam & Burns 2012), financial insecurity (Hartley *et al*. 2011; Ross *et al*. 2009), and low or moderate ‘satisfaction with ability to access basic needs’ (Kaaya, Mbwambo, Kilonzo, Van Den Borne, Leshabari, Fawzi, *et al*. 2010). Health-related risk factors associated with depression include poorer physical health (Kaida *et al*. 2014; Ross *et al*. 2009) and having had a previous depressive episode (Kaaya *et al*. 2010; Manikkam & Burns 2012). Psychosocial variables, such as low self-esteem (Ross *et al*. 2009), perceived stress (Blaney, Fernandez, Ethier, Wilson, Walter, Koenig & Perinatal Guidelines Evaluation Project Group 2004), social isolation (Blaney *et al*. 2004), lack of emotional support (Ross *et al*. 2009), lower perceived social support (Stewart, Umar, Tomenson & Creed 2014), lack of partner support (Hartley *et al*. 2011), disengagement coping (Blaney *et al*. 2004; Kotzé, Visser, Makin, Sikkema & Forsyth 2013a), unplanned pregnancy (Manikkam & Burns 2012), conflicts with the current partner (Kaaya *et al*. 2010), and experience of intimate partner violence (IPV; Hartley *et al*. 2011; Stewart *et al*. 2014) have also been associated with depressive symptomatology. With regard to treatment, poor antiretroviral (ARV) medication adherence (Nachega, Uthman, Anderson, Peltzer, Wampold, Cotton, *et al*. 2012; Sheth, Coleman, Cannon, Milio, Keller, Anderson, *et al*. 2015), low utilization of prenatal care (Psaros, Geller & Aaron 2009), decreased time on antiretroviral therapy (ART; Kaida *et al*. 2014) and poor treatment outcomes (Kaida *et al*. 2014; Sheth *et al*. 2015) have been found to be associated with depressive symptoms.

In a recent review of the literature on the mental health outcomes of HIV-positive pregnant women in South Africa (Kapetanovic *et al*. 2014), only six studies were identified. Of these, five (Kotzé *et al*. 2013a, 2013b; Manikkam & Burns 2012; Mfusi & Mahabeer 2000; Mundell, Visser, Makin, Kershaw, Forsyth, Jeffery, *et al*. 2011) assessed perinatal and postnatal depression. However, only one of these specifically sought to assess the prevalence, risk factors and psychosocial correlates of prenatal depression among HIV-positive women in an urban area of South Africa (Manikkam & Burns 2012). The identified correlates of depression in that study included HIV seropositivity, history of depression, suicidality, being single, and having an unplanned pregnancy. However, none of these studies have addressed the risk factors and psychosocial correlates of depression among HIV-positive pregnant women in rural South Africa. Examining the prevalence and associated factors of depression in rural areas may help identify population-specific risk factors in this group of women. Therefore, the present study aimed to assess the prevalence of depressive symptoms and associated factors in prenatal HIV-positive women in community healthcare centre facilities in Mpumalanga province, South Africa.

## Method

### Participants and procedures

Participants were 663 HIV-positive pregnant women in South Africa recruited during the baseline phase of Protect Your Family, a clinic-randomized controlled trial designed to test the effectiveness of a behavioural intervention aimed at increasing prevention of mother to child transmission (PMTCT) uptake, family planning and male partner participation in the antenatal and postnatal process in 12 randomly selected community health centres in Gert Sibande and Nkangala districts in Mpumalanga province, South Africa (Jones, Peltzer, Weiss, Sifunda, Dwane, Ramlagan, *et al*. 2014).

Both newly diagnosed and previously diagnosed women were recruited using systematic sampling. Newly diagnosed women had received pre- and post-HIV counselling and testing (HCT) per South African PMTCT protocol and referral for immediate CD4 assessment and ART. As such, study-eligible candidates were referred to the external study assessor by PMTCT/HCT staff post-HIV testing and referral for treatment. Given the study design, only seropositive women with partners were approached to participate in the study, although their partners were not enrolled in the study. Additional inclusion criteria included being 20–24 weeks pregnant, the typical time of entry into antenatal care, and being aged 18 or older. Potential participants who did not meet these criteria were not allowed to participate. Interested and eligible participants were offered an appointment, and enrolled after provision of informed consent.

After enrolment, all women completed study measures in their preferred language (English, Zulu, or Sotho) using Questionnaire Development System Audio Computer-Assisted Self-Interview software in order to enhance disclosure, accommodate all levels of literacy and reduce interviewer bias. To familiarize participants with the software, assessors completed the demographic component of the questionnaire with participants prior to completion of all other assessments. In addition, an on-site assessor was available at all times to answer any questions.

A physician, clinic officer, counsellor or other trained healthcare provider at each clinic was available to immediately evaluate participants who disclosed experiencing serious depression or suicidality. After assessing the level of risk, if so deemed by the provider, the participant was referred for further assessment and/or hospitalization, or, if not in immediate danger of harming herself, for outpatient counseling/treatment. All research sites had access at all times to licensed psychiatric nurses and social workers, as well as psychiatrists and clinical psychologists at the district hospital.

Prior to the onset of study activities, approval was obtained from the institutional ethics committee, the institutional review board and local health authorities (provincial, district, sub-district and clinic level). The Protect Your Family trial protocol has been previously published (Jones *et al*. 2014) and is registered on clinicaltrials.gov, number NCT02085356. Sample size for this descriptive study, expecting a prevalence of 50% of depressive symptoms (Kaida *et al*. 2014; Kwalombota 2002; Natamba *et al*. 2014; Ross *et al*. 2009; Smith *et al*. 2011), was calculated by using Software Epi-Info Version 7, and gave a sample size of 663, for population survey, confidence limits 5% and confidence level 99%.

#### Measures

##### Sociodemographics, and HIV-related questions

Participants responded to demographic questions, including age, education, income, employment status, number of children, planned pregnancy, alcohol use, condom use at last sex, time since HIV diagnosis, and time since ART initiation.

##### Depression

The Edinburgh Postnatal Depression Scale 10 (EPDS-10; Cox, Holden & Sagovsky 1987) was used to assess depression, adapted for perinatal depression. The EDPS-10 is a 10-item instrument asking participants to rate how often they have experienced different symptoms associated with depression in the past 7 days. Scores range from 0 through 30; the validated cut-off score for South African populations is 12 (Lawrie, Hofmeyr, de Jager & Berk 1998). Cronbach alpha for the EDPS-10 scale was 0.80 in this study sample, showing adequate internal consistency, as found in prior research in South Africa (Peltzer & Shikwane 2011).

##### Stigma

The AIDS-Related Stigma Scale (Kalichman, Simbayi, Jooste, Toefy, Cain, Cherry, *et al* 2005) is a nine-item subscale that measures stigma, for example, ‘People who have AIDS should be ashamed,’ which are rated dichotomously using either a score of 0 (*Disagree*) or 1 (*Agree*). Therefore, scores on this scale range from 0 to 9, where higher scores indicate greater levels of stigma. The reversed coded item for this scale (‘It is safe for people who have AIDS to work with children’) was excluded given the scale’s poor internal reliability (*α* = 0.58) with its inclusion. Excluding the item, reliability was adequate (*α* = 0.74). Scores 0 and scores of 1 through 8 were dichotomized into 0 and 1, respectively.

##### Disclosure

HIV disclosure was assessed using an adaptation of the Disclosure Scale (Visser, Neufeld, De Villiers, Makin & Forsyth 2008), which assesses disclosure among sexual partners and family members during pregnancy and factors associated with disclosure.

##### Intimate partner violence

IPV was assessed using an adaptation of Conflict Tactics Scale 18 (CTS-18; Straus 1979), which assesses components of conflict resolution as subscales of reasoning, psychological aggression, mild and severe physical aggression. Respondents indicate the number of times in the past 6 months their partner has engaged in specific behaviours using a scale of 0 (Never) to 6 (More than 20 times); for the purposes of this study, participants were asked to respond to queries referencing the past 4 weeks, as this would reflect IPV during pregnancy. Furthermore, in this study, only the severe physical aggression subscale total was considered, and were dichotomized into a score of 0 if they reported no severe physical aggression, and 1 if they reported any form of severe physical aggression.

##### Number of skips in past week

Participants stated the number of times they skipped a dose of their ARV medication in the past week. Participant responses endorsing no skipped medication in the past week were dichotomized into a score of 0; responses were scored as 1 if reporting skipping medication in the past week.

##### Male involvement

Male involvement was assessed using a Male Involvement Index (Jones *et al*. 2014), comprised of 11 items related to the participant’s partner’s involvement in the antenatal period. Questions included ‘Does your male partner attend antenatal care visits with you?’ and ‘Have you discussed antenatal HIV prevention for your baby with your male partner?’ Participants responded to each item as 1 (Yes) or 0 (No), and scores ranged from 0 to 11. Cronbach’s alpha was 0.82 in this sample.

#### Data analysis

The Statistical Package for Social Sciences (SPSS version 18.0 for Windows; SPSS Inc., Chicago, IL, USA) was used for data analyses. Descriptive data on the total sample were first examined, and bootstrap confidence intervals for the prevalence of depression and response rate were calculated using 1000 bootstrap samples. Prenatal women were then classified as having depressed mood or not, based on a score greater than or equal to 13 on the EPDS-10. Bivariate analysis and multivariable logistic regressions were used to investigate associations between the socio-demographic, alcohol use, HIV- and partner-related variables and depressed mood. Significant associations in bivariate analysis were included in a multivariable model using a backward elimination procedure. Variables not significant at *p* > .05 were excluded from the final model. Lastly, possible two-way interactions were tested in the reduced model, and a final model with an interaction term was built using a sequential logistic regression analysis to test the reduced model against a model with an interaction term. Contrasts were used to follow up a significant interaction effect. Associations were considered significant at *p* < .05.

## Results

### Sample characteristics

Of the 709 HIV-positive pregnant women invited to participate in the study, eight declined, and 38 had unusable data resulting in a total sample of 663 participants (response rate 93.5% [95% CI: 91.7, 95.1]). The mean age of the women was 28.3 (SD = 5.7) years, with a range of 18–46 years. The majority (79.2%) of the participants had one or more children. Twenty-nine per cent had 12 or more years of formal education, 49.5% 10–11 years and 22.0% 9 years or less education. Most pregnant women (83.1%) were not employed, and two-thirds (67.0%) had a monthly income of less than 950 South African Rand.

For more than half (52.9%) the current pregnancy had not been planned. A small group of women (13.7%) reported that they drink two or more alcoholic beverages on at least one occasion in the past month. More than half (54.1% [95% CI: 50.2, 57.9]) had been diagnosed with HIV in their current pregnancy, and most (72.1%) had disclosed their HIV status to someone, and 58.5% to their partner. Among those women who had children, 5.1% knew that they had an HIV-positive child. Two-thirds (67.1%) had not skipped any of their medication in past week. Almost half of the women (47.8%) had not used a condom at their last sexual intercourse, and 19.6% reported to have experienced mild or severe physical partner violence in the past 4 weeks. In all, 48.7% [95% CI: 44.8, 52.6] scored ≥ 13 on the EDPS-10 (see Tables 1 and 2).

**Table 1.** Sample characteristics in terms of sociodemographics and alcohol use of HIV-positive pregnant women by depressive symptoms status (*N* = 663).

Variables	All (*N* = 663)	Depressive symptoms (*n* = 323)	Non-depressive symptoms (*n* = 340)	Statistic
	*N* (%) Mean (SD)	*N* (%) Mean (SD)	*N* (%) Mean (SD)	*t*/*χ*^2^, *p*
Sociodemographics and alcohol use				
All	663	323 (48.7%)	340 (51.3%)	
Age	28.3 (5.7)	28.3 (5.8)	28.4 (5.7)	.200; .841
Educational attainment				
<Grade 10	146 (22.0%)	80 (24.8%)	66 (19.4%)	11.14; .004
Grade 10–11	328 (49.8%)	170 (52.6%)	158 (46.5%)	
Grade 12 or more	189 (28.5%)	73 (22.6%)	116 (34.1%)	
Employment status				
Not employed	551 (83.1%)	280 (86.7%)	271 (79.7%)	5.75; .016
Employed	112 (16.9%)	43 (13.3%)	69 (20.3%)	
Income (South African Rand)				
<310	220 (33.2%)	111 (34.4%)	109 (32.1%)	7.29; .026
310–949	224 (33.8%)	121 (37.5%)	103 (30.3%)	
950 or more	219 (33.0%)	91 (28.2%)	128 (37.6%)	
Number of children				
None	138 (20.8%)	64 (19.8%)	74 (21.8%)	.474; .789
One	260 (39.2%)	130 (40.2%)	130 (38.2%)	
Two or more	265 (40.0%)	129 (39.9%)	136 (40.0%)	
Pregnancy planned				
Yes	312 (47.1%)	129 (39.9%)	183 (53.8%)	12.82; <.001
No	351 (52.9%)	194 (60.1%)	157 (46.2%)	
Alcohol use of 2 or more drinks at least once in the past 4 weeks				
No	572 (86.3%)	271 (83.9%)	301 (88.5%)	3.00; .083
Yes	91 (13.7%)	52 (16.1%)	39 (11.5%)

**Table 2.** Sample characteristics in terms of HIV- and partner-related variables of HIV-positive pregnant women by depressive symptoms status (continued).

Variables	All (*N* = 663)	Depressive symptoms (*n* = 321)	Non-depressive symptoms (*n* = 338)	Statistic
	*N* (%) Mean (SD)	*N* (%) Mean (SD)	*N* (%) Mean (SD)	*t*/*χ*^2^, *p*
**HIV-related variables**				
Diagnosed with HIV in this pregnancy				
No, before	304 (45.9%)	146 (45.2%)	158 (46.5%)	.107; .743
Yes	359 (54.1%)	177 (54.8%)	182 (53.5%)	
Disclosure of HIV serostatus to anyone				
No	185 (27.9%)	98 (30.3%)	87 (25.6%)	1.86; .173
Yes	478 (72.1%)	225 (69.7%)	253 (74.4%)	
Months since ART initiation	13.0 (23.9)	11.8 (22.3)	14.0 (25.3)	1.09; .277^a^
Has HIV-positive children				
No or do not know	498 (94.9%)	252 (97.3%)	246 (92.5%)	6.23; .012
Yes	27 (5.1%)	7 (2.7%)	20 (7.5%)	
Internalized AIDS stigma				
Score 0	391 (59.0%)	173 (53.6%)	218 (64.1%)	7.63; .006
Score 1 or more	272 (41.0%)	150 (46.4%)	122 (35.9%)	
Skipped medication in the past 7 days				
Non-adherent (<100%)	218 (32.9%)	132 (40.9%)	86 (25.3%)	18.20; <.001
Adherent (100%)	445 (67.1%)	191 (59.1%)	254 (74.6%)	
**Partner variables**				
Disclosure of HIV serostatus to partner				
No	275 (41.5%)	145 (44.9%)	130 (38.2%)	3.02; .082
Yes	388 (58.5%)	178 (55.1%)	210 (61.8%)	
HIV-positive partner				
No or do not know	497 (75.0%)	253 (78.3%)	244 (71.8%)	3.80; .051
Yes	166 (25.0%)	70 (21.7%)	96 (28.2%)	
Condom use at last sex				
Yes	317 (47.8%)	176 (54.5%)	141 (41.5%)	11.25; <.001
No	346 (52.2%)	147 (45.5%)	199 (58.5%)	
IPV				
No mild or severe physical violence	533 (80.4%)	239 (74.0%)	294 (86.5%)	16.36; <.001
Mild or severe physical violence	130 (19.6%)	84 (26.0%)	46 (13.5%)	
Male involvement index	7.15 (3.0)	6.7 (3.2)	7.6 (2.8)	3.58; <.001

^a^Mann–Whitney U test was used for comparison.

### Associations with depressive symptoms

In bivariate analysis, less education, unemployment, unplanned pregnancy, not having an HIV-positive child, increased internalized AIDS stigma, ART non-adherence, non-condom use at last sex, IPV and lack of male involvement were associated with depressive symptoms. In multivariate analysis, unemployment, unplanned pregnancy, not having an HIV-positive child, ART non-adherence, IPV and lack of male involvement were associated with depressive symptoms (see Table 3). In the final model, there was a significant interaction between IPV and unplanned pregnancy (Table 4). Pairwise contrasts were used to follow the interaction effect, which indicated that among woman reporting IPV, the odds of unplanned pregnancy predicting depression were higher than that of planned pregnancy (*p* < .001), but when women did not report IPV, there was no difference in the odds of depression between unplanned versus planned pregnancy (*p* > .05).

**Table 3.** Associations between sociodemographic, alcohol use, HIV-related and partner-related variables and depressive symptoms.

Variables	Unadjusted OR (CI = 95%)	Adjusted OR (CI = 95%)^a^
**Sociodemographics and alcohol use**		
Age	1.00 (0.97–1.02)	NS
Educational attainment		
<Grade 10	1.00	NS
Grade 10–11	0.89 (0.60–1.31)	
Grade 12 or more	0.52 (0.34–0.85)**	
Employed (reference = not employed)	0.60 (0.40–0.91)*	0.57 (0.37–0.88)**
Income (South African Rand)		
<310	1.00	NS
310–949	1.15 (0.80–1.68)	
950 or more	0.70 (0.48–1.02)	
Number of children		
None	1.00	NS
One	1.16 (0.77–1.77)	
Two or more	1.10 (0.73–1.66)	
Pregnancy unplanned (ref = planned)	1.75 (1.29–2.39)***	1.69 (1.22–2.33)**
Whether participant has had more than 2 drinks on one occasion in the past 4 weeks (ref = no)	1.48 (0.95–2.31)	NS
**HIV-related variables**		
Diagnosed with HIV in this pregnancy (ref = no)	1.05 (0.78–1.43)	NS
Disclosure of HIV serostatus to anyone (ref = no)	0.79 (0.56–1.11)	NS
Months since ART initiation	0.996 (0.99–1.003)	NS
HIV-positive children (ref = no or do not know)	0.34 (0.14–0.82)*	0.28 (0.11–0.71)**
Internalized AIDS stigma (score 1 or more) (ref = 0)	1.55 (1.14–2.12)*	NS
Adherence (ref = nonadherent)	0.49 (0.35–0.68)***	0.51 (0.36–0.72)***
**Partner variables**		
Disclosure of HIV serostatus to partner (ref = no)	0.78 (0.49–1.23)	NS
HIV-positive partner (ref = no or do not know)	1.42 (0.997–2.03)	NS
Non condom use at last sex (ref = yes)	1.69 (1.24–2.30)**	1.47 (1.05–2.06)*
IPV (ref = no)	2.25 (1.51–3.34)***	1.99 (1.30–3.03)**
Male involvement index	0.91 (0.86–0.95)***	0.93 (0.88–0.9)*

Note: NS = not significant in multivariable model; ART = antiretroviral therapy.

^a^Hosmer and Lemeshow chi-square = 25.44, *p* < .05; Nagelkerke *R*^2^ = 0.14.

***P < .001.

**P < .01.

*P < .05.

**Table 4.** Final model of associations between sociodemographic, alcohol use, HIV-related and partner-related variables and depressive symptoms.

Variables	*b* (SE)	OR (CI = 95%)^a^
Pregnancy unplanned (ref = planned)	0.69 (0.28)	1.98 (1.38–2.83)***
IPV (ref = no)	1.19 (0.34)	3.30 (1.70–6.38)***
IPV × pregnancy unplanned	−0.89 (0.43)	0.42 (0.18-.97)*
Employed (reference = not employed)	−0.55 (0.22)	0.57 (0.37–0.88)*
HIV-positive children (ref = no or do not know)	−1.16 (0.47)	0.31 (0.12–0.79)*
Adherence (ref = nonadherent)	−0.70 (0.18)	0.51 (0.36–0.71)***
Male involvement index	−0.09 (0.03)	0.93 (0.88–0.99)*

Note: NS = not significant in multivariable model; ART = antiretroviral therapy.

^a^Hosmer and Lemeshow chi-square = 12.95, *p* > .05; Nagelkerke *R*^2^ = .15.

***P < .001.

**P < .01.

*P < .05.

## Discussion

This study found a high prevalence of depressive symptoms (48.7% [95% CI: 44.8, 52.6]) in this sample of pregnant HIV-infected women in healthcare facilities in rural South Africa. Risk factors and correlates of depression appeared associated with both demographic and psychosocial factors. Study outcomes have important implications for public health intervention.

Results of this study may primarily reflect the experience of living in poverty in rural South Africa. Not surprisingly, income and education were associated with depression in a setting where unemployment is the norm and many women are forced to leave school to seek work due to poverty (Tolla 2013). For most, the current pregnancy was unexpected and resources to support the child may be limited; the association with depression is not unlike that obtained in earlier studies (urban Cape Town, Hartley *et al*. 2011; Uganda, Kaida *et al*. 2014; Zambia, Kwalombota 2002; urban KwaZulu-Natal, Manikkam & Burns 2012; Natamba *et al*. 2014; Thailand, Ross *et al*. 2009; Tanzania, Smith Fawzi *et al*. 2007). Similarly, the association of depression with violence has been noted in previous studies (Hartley *et al*. 2011; Kaaya *et al*. 2010; Stewart *et al*. 2014). These findings suggest that partner dynamics may influence the well-being of HIV-positive mothers. For women in this sample, living in poverty with HIV infection and for some, experiencing violence, pregnancy may be an unwelcome addition to existing burdens. These potentially population-specific risk factors have important implications for both mothers and their unborn children, many of whom may be unable to take medication to prevent HIV transmission due to lack of HIV disclosure. As found in previous studies (Nachega *et al*. 2012; Sheth *et al*. 2015), one third of this sample endorsed non-adherence to ART, and non-adherence was associated with depressive symptoms. Maternal depression has been associated with suboptimal child development (Canadian Paediatric Society 2004). Study outcomes suggest the need for ongoing depression screening and referral systems for HIV-infected women during pregnancy.

Unexpectedly, having an HIV-infected child was found to be protective against depressive symptoms in this sample. In contrast to previous research (Zambia; Kwalombota 2002), women diagnosed with HIV in the current pregnancy, compared to those who had been previously diagnosed, were not more likely to report depressive symptoms, despite the potentially negative association between lack of social support and depressive symptoms (Ross *et al*. 2009; Stewart *et al*. 2014). It is possible that having an infant with HIV may foster a sense of belongingness and social support in this group of women, which has been found in South Africa to be a protective factor against depression (Cooper, Tomlinson, Swartz, Woolgar, Murray & Molteno 1999).

Decreased male involvement was associated with depression in multivariable analysis in this study; lack of partner support among pregnant women has been associated with depression in a previous study in South Africa (Hartley *et al*. 2011). The association between decreased partner support and maternal depression (pre- and postnatally) is consistent with previous studies (Pajuloa, Savonlahtia, Sourandera, Heleniusb & Pihaa 2001; Ramchandani, Richter, Stein & Norris 2009; Stapleton, Schetter, Westling, Rini, Glynn, Hobel, *et al*. 2012; Tomlinson, Swartz, Cooper & Molteno 2004). In this study, male partner involvement was assessed in terms of antenatal care attendance (visit, appointment time, discussion, financial support) and HIV issues (discussion, partner testing, disclosure, ARV use, infant feeding). It has been suggested that partner support during pregnancy may lead to better maternal and neonatal outcomes, which provides an opportunity for enhanced mental health to be associated with PMTCT interventions (Stapleton *et al*. 2012). Given the potential benefits of male involvement on maternal health and infant development, it has been recommended the South African PMTCT protocol should highlight the importance of male partner involvement, as well as provide specific plans on promoting and increasing male involvement (Van den Berg, Brittain, Mercer, Peacock, Stinson, Janson, *et al*. 2015).

Unlike in previous studies, internalized AIDS stigma was associated with depressive symptoms in univariate analysis (Peltzer & Shukwane 2011; Wight 2000), but not when accounting for other variables. This suggests non-disclosure to partners may be a potential contributor to this issue. Further research is needed to examine the impact of male involvement and disclosure to male partners on pregnancy outcomes among depressed HIV-positive women.

### Study strength and limitations

The study utilized a large sample of HIV-positive pregnant women assessed using a uniform methodology across 12 health facilities in two geographically different health districts. It is not known whether these results are generalizable to other areas and populations. Study participants were limited to HIV-positive pregnant women having male partners. It is possible that different depression prevalence rates would be found among women without current male partners in comparison with women with male partners. The study also relied on self-report of depression not verified by a diagnostic interview, and may under- or over report depression. In addition, cross-sectional examination prevents any causal inferences; longitudinal analysis may provide additional insight into maternal depression by clarifying temporal associations. Finally, certain factors that have been found to be associated with depressive symptoms in previous studies were not assessed in this study and should be included in future studies, such as physical health status (Kaida *et al*. 2014; Ross *et al*. 2009), having an existing history of depression (Kaaya *et al*. 2010; Manikkam & Burns 2012), perceived stress (Blaney *et al*. 2004), coping style (Blaney *et al*. 2004; Kotzé *et al*. 2013a), utilization of prenatal care (Psaros *et al*. 2009) and treatment outcome (Sheth *et al*. 2015).

## Conclusion

This study was conducted in 12 community health centres in two health districts in Mpumalanga Province, and results provide new information regarding the mental health status of pregnant HIV-positive women, identifying clinical and psychosocial correlates for potential consideration in the development and implementation of interventions targeting this group. The study found a high prevalence of depression and several associated factors of prenatal depressive symptoms among HIV-positive pregnant women. The development of targeted interventions, that is, preconception counselling to help plan pregnancies, encouraging partner involvement campaigns, focusing on adherence and reasons for non-adherence and training health workers to address partner violence, could specifically respond to these factors. We recommend that screening for prenatal depression and access to mental health interventions should be part of routine antenatal care for all women, and join existing investigators (Stringer, Meltzer-Brody, Kasaro, Stuebe, Wiegand, Paul, *et al*., 2014) who promote the incorporation of the Edinburgh Postnatal Depression Scale or a similar depression-screening technique in the antenatal setting, during HIV pre-test or post-test counselling activities.
